# Comparing the factor structures and reliabilities of the EPDS and the PHQ-9 for screening antepartum and postpartum depression: a multigroup confirmatory factor analysis

**DOI:** 10.1007/s00737-023-01337-w

**Published:** 2023-07-18

**Authors:** Alberto Stefana, Joshua A. Langfus, Gabriella Palumbo, Loredana Cena, Alice Trainini, Antonella Gigantesco, Fiorino Mirabella

**Affiliations:** 1 Department of Brain and Behavioral Sciences, University of Pavia, Pavia, Italy; 2 Department of Psychology and Neuroscience, University of North Carolina at Chapel Hill, Chapel Hill, USA; 3 Center for Behavioural Sciences and Mental Health, National Institute of Health, Rome, Italy; 4 Department of Clinical and Experimental Sciences, Section of Neuroscience, Observatory of Perinatal Clinical Psychology, University of Brescia, Brescia, Italy

**Keywords:** EPDS, PHQ-9, Antenatal depression, Postnatal depression, Screening, Postpartum people, Pregnant people

## Abstract

To evaluate and compare the factor structure and reliability of EPDS and PHQ in antepartum and postpartum samples. Parallel analysis and exploratory factor analysis were conducted to determine the structure of both scales in the entire sample as well as in the antepartum and postpartum groups. McDonald’s omega statistics examined the utility of treating items as a single scale versus multiple factors. Multigroup confirmatory factor analysis (MCFA) was utilized to test the measurement invariance between the antepartum and postpartum groups. Two-factor models fit best for the EPDS in both the antepartum and postpartum groups; however, the most reliable score variance was attributable to a general factor for each scale. MCFA provided evidence of weak invariance across groups regarding factor loadings and partial invariance regarding item thresholds. PHQ-9 showed a two-factor model in the antepartum group; however, the same model did not fit well in the postpartum group. EPDS should be preferred to PHQ-9 for measuring depressive symptoms in peripartum populations. Both scales should be used as a single-factor scale. Caution is required when comparing the antepartum and postpartum scores.

## Introduction

Perinatal depression is a unipolar, non-psychotic depressive disorder (Howard et al. 2014) characterized by specific feelings and thoughts about the parental role (Langan & Goodbred 2016). It is one of the leading complications for people during pregnancy (antepartum depression) or following childbirth (postpartum depression) (Howard et al. 2014; Howard & Khalifeh 2020). Recent meta-analyses of maternal depression observed a pooled prevalence of 15% and 14%, respectively, for pre- and postnatal depression (Liu Wang & Wang 2022; Yin et al. 2021).

Both new-onset and preexisting depression in pregnant or postpartum people are associated with increased maternal mortality, suicide, and self-harm, as well as adverse obstetrical, neonatal, and long-term outcomes for children (Howard & Khalifeh 2020). All of these consequences lead to substantially increased costs for healthcare systems (Knapp & Wong 2020; Luca et al. 2020). However, growing evidence has identified early screening and prompt management as crucial factors in reducing symptoms and preventing relapses in perinatal people and their families (Austin et al. 2017; O’Connor et al. 2019; Cena et al. 2021).

In line with the World Health Organization’s (2020) recommendations, routine screening for perinatal depression through valid, reliable, and economical screening tools is probably the most widely accepted suggestion (Accortt & Wong 2017; ACOG Committee 2018). However, a consensus has not been reached on what scale can be considered the gold standard. The most frequently validated and utilized screening tools are the Edinburgh Postnatal Depression Scale (EPDS), Beck’s Depression Inventory (BDI), and the Patient Health Questionnaire (PHQ-9) (Sambrook Smith et al. 2022a,b). Differently from BDI, both the EPDS and PHQ-9 are free to use as public domain measures.

On a theoretical level, a well-developed and validated disease-specific questionnaire should measure the same construct across different settings and patient populations. Based on this premise and given the clinical and research needs, the PHQ-9 and EPDS are widely used to evaluate levels of depressive symptomatology in both pregnant and postpartum people, examine their developmental trajectories, and compare the results among different groups. However, previous studies have found inconsistent factor structures for both the PHQ-9 and EPDS depending on the perinatal period (i.e., antepartum versus postpartum), ranging from one-factor (e.g., Berle et al. 2003; Woldetensay et al. 2018) to three-factor solutions (e.g., Marcos-Nájera et al. 2018; Matsumura et al. 2020). It should be noted that very few studies have investigated the factor structure of the PHQ-9 with pregnant people and, as far as we know, no study has investigated it in a postpartum sample or tested the measurement invariance of this measure across the perinatal period. Furthermore, regarding the Italian version of both of these two scales, only the EPDS was validated in a perinatal sample (more specifically, a postpartum sample).

Therefore, the aim of the present study is to evaluate and compare the factor structure and reliability of both the EPDS and the PHQ in antepartum versus postpartum samples and test for measurement invariance across the perinatal period.

## Methods

### Study design and sample

The data presented here were collected as baseline data for a longitudinal study (March 2017–June 2018) on screening and early intervention for maternal perinatal anxiety and depressive disorders. Eleven publicly funded primary or obstetrics-gynecology secondary care centers located throughout Italy were involved in the study as recruitment sites. The inclusion criteria were being pregnant regardless of the trimester of pregnancy (antepartum group) or having a biological newborn aged ≤6 months (postpartum group), and being able to speak and read Italian. The exclusion criteria were having issues with drug or substance misuse and/or having ongoing psychotic symptoms. All participants signed informed consent forms after being provided oral and written explanations of the aims and protocol of this study. This study was approved by the ethics committee of the Ethical Committee of the Healthcare Centre of Bologna Hospital. The rationale and full methodology of the larger study have been described in the study protocol (Cena et al. 2020).

### Data collection

Each participant was interviewed in a private room inside the healthcare center by a clinical psychologist trained in perinatal clinical psychology and associated with the healthcare center. The aim of the interview was to gather information on the participants’ current and past psychiatric conditions and the use of psychotropic drugs, as well as their current experience with symptoms of stress, anxiety, and depression. At the end of that interview session, all participants completed the EPDS and PHQ-9 themselves as self-audit. Information on the demographic, economic, and psychosocial as well as reproductive characteristics of participants was collected.

### Measures

#### Edinburgh Postnatal Depression Scale

The EPDS (Cox et al. 1987) is the most widely used self-administered instrument to screen for perinatal depression (Sambrook Smith et al. 2022a,b). It can be used to assess depression according to the DSM-5 (American Psychiatric Association 2013) criteria (Smith-Nielsen et al. 2018). The EPDS was originally designed to assess the severity of depressive symptoms in new mothers and was subsequently used to screen for antepartum depression. It assesses the frequency of each of the following depressive symptoms as experienced in the previous 7 days: anhedonia (two items), guilt, anxiety, panic attack, feeling overwhelmed, sleep disturbance, sadness, tearfulness, and suicidal thoughts. The validated Italian translation of the EPDS showed a Cronbach alpha coefficient of 0.79 and a Guttman split-half coefficient of 0.81 (Benvenuti et al. 1999).

#### Patient Health Questionnaire-9

The PHQ-9 (Kroenke et al. 2001) is a self-administered depression screening scale containing nine items corresponding to the DSM-IV (Association American Psychiatric 1994) criteria for depression. Furthermore, it can measure depression severity based on the DSM-5 (American Psychiatric Association 2013) criteria (Spitzer et al. 2014). The PHQ-9 is the most widely used depression measure across clinical practice settings worldwide (Hirschtritt & Kroenke 2017; Kroenke 2021) and has been identified as the most reliable depression screening tool (El-Den et al. 2018; Negeri et al. 2021). It assesses the frequency of each of the following depressive symptoms as experienced in the previous 2 weeks: anhedonia, depressed mood, insomnia or hypersomnia, fatigue or loss of energy, appetite disturbances, feelings of worthlessness or excessive guilt, diminished ability to think or concentrate, psychomotor agitation or retardation, and suicidal thoughts. The internal consistency (Cronbach’s alpha) of the PHQ-9 administered to an obstetric−gynecology sample was 0.86 (Kroenke et al. 2001). The Italian translation of the PHQ-9 showed sensitivity, specificity, and positive predictive values of 39, 29, and 93%, respectively, for any depressive syndrome (Mazzotti et al. 2003).

### Statistical analyses

Descriptive statistics were computed for each variable, including means and standard deviations (SDs) for continuous variables and frequencies and percentages for categorical variables. Parallel analysis using the R package *EFAtools* v0.4.1 (Steiner & Grieder 2020) was performed on a polychoric correlation matrix using the mean eigenvalues and 95th percentile eigenvalues of 5,000 simulated random datasets. The factor structures of both the EPDS and PHQ-9 were explored separately through exploratory factor analysis (EFA) and multiple-group confirmatory factor analysis (CFA) using the R packages *EFAtools* v0.4.1 (Steiner & Grieder 2020) and *lavaan* v0.6-11 (Rosseel, 2012). First, parallel analysis evaluated the number of factors that may be supported by the data in the entire sample as well as in the antepartum and postpartum subgroups by comparing actual eigenvalues to random eigenvalues sampled at the 95th percentile (Glorfeld 1995). Scree plots were also examined. The scree plot and eigenvalues associated with each factor were also used to identify the number of meaningful factors. Next, a series of EFA models with maximum likelihood extraction and oblique rotation was performed to evaluate item loadings. These analyses were repeated three times, setting the extracted number of the factors to three, two, and one given the results of parallel analyses and also because no studies indicated structures of four or more factors for both the EPDS and PHQ-9 (for a review of various factor models of the EPDS see Matsumura et al. 2020; for the PHQ-9 see Barthel et al. 2015; Smith et al. 2022a,b; and Marcos-Nájera et al. 2018). Factor loadings ≥ 0.32 were used in the factor designation (Tabachnick & Fidell 2019). Next, the model with the best fit was tested by the multiple-group CFA method in order to assess measurement invariance between pre- and postnatal groups. A well-fitting baseline model was established, and the effects of equality constraints across groups were evaluated by likelihood ratio tests. Evidence for reasonably good fit was assessed using standard fit indices, including the root mean square error of approximation (RMSEA; values close to 0.06 or below are considered good) and comparative fit index (CFI; close to 0.95 or greater). All tests were two-tailed, with the statistical significance level set at *α* = 0.05. Lastly, omega reliability coefficients were calculated using the R package *Psych* v2.2.9 (Revelle 2022). Omega total measures the total reliable variance for each scale, and omega hierarchical indexes the variance attributable to a single general factor. High values of omega total indicate an overall reliable scale, and high omega hierarchical values support interpreting item scores as a single scale.

All statistical analyses were performed with R version 4.2.0 (R Core Team 2022).

## Results

### Sample characteristics

Approximately 30% of the subjects approached refused to participate in the study, and *n* = 1 subject was not eligible to participate due to ongoing psychotic symptoms. No participants dropped out during the baseline evaluation. The overall sample included 1477 people: 1166 pregnant people and 311 new mothers. The two groups did not differ in nationality, marital status, educational level, working status, economic status, having planned the pregnancy or not, resorting to assisted reproductive technology or not, and history of past abortions. Compared to pregnant people, new mothers were older (*p* < 0.01), were more likely to have previous pregnancies (*p* < 0.01), and had children living at the time of this pregnancy/birth (*p* < 0.01). The sociodemographic and reproductive information are shown in Table 1.

**Table 1:** Sociodemographics and reproductive characteristics of the sample

	Antepartum sample	Postpartum sample	Entire sample
Total	*n* (%)	*n* (%)	*N* (%)
Age**			
18–29	278 (23.9)	51 (16.5)	329 (22.3)
30–35	545 (46.8)	135 (43.5)	680 (46.2)
> 35	341 (29.3)	124 (40.0)	465 (31.5)
Marital status			
Married or cohabiting	1060 (91.7)	278 (90.3)	1338 (91.4)
Single, separated, divorced, or widowed	96 (8.3)	30 (9.7)	126 (8.6)
Educational level			
University	595 (51.5)	139 (45.3)	734 (50.2)
Secondary	417 (36.0)	119 (38.7)	536 (36.6)
Primary or illiterate	144 (12.5)	49 (16.0)	193 (13.2)
Working status			
Permanent employee	829 (72.0)	210 (68.4)	1039 (71.3)
Temporary employee	117 (10.2)	25 (8.1)	142 (9.7)
Student, homemaker, or unemployed	205 (17.8)	72 (23.5)	277 (19.0)
Economic Status			
Average high status	529 (46.0)	123 (40.2)	652 (44.7)
A few problems without specific difficulties	547 (47.5)	155 (50.6)	702 (48.2)
Same or many problems	75 (6.5)	28 (9.2)	103 (7.1)
Planned pregnancy			
Yes	816 (70.9)	236 (76.4)	1052 (72.1)
No	335 (29.1)	73 (23.6)	408 (27.9)
Previous pregnancies **			
Yes	291 (25.0)	132 (42.4)	423 (28.6)
No	875 (75.0)	179 (57.6)	1054 (71.4)
Past abortion(s)			
Yes	301 (26.1)	91 (29.7)	392 (26.9)
No	851 (73.9)	215 (70.3)	1066 (73.1)
Children living at the time of this pregnancy/birth**			
Yes	195 (16.7)	115 (37.0)	310 (21.0)
No	971 (83.3)	196 (63.0)	1167 (79.0)

The antepartum sample consists of 1166 pregnant people, while the postpartum sample consists of 311 people who gave birth to one or more children in the 6 months prior to the time of data collection. The entire sample includes both antepartum and postpartum samples

*p < 0.05; **p < 0.01

### Parallel analysis

The number of factors identified by the parallel analyses with principal component analysis (PCA), exploratory factor analysis (EFA), and squared multiple correlation (SMC) was as follows: EPDS whole group: one, five, and six; EPDS antepartum group: two, six, and six; EPDS postpartum group: one, four, and NA; PHQ-9 whole group: one, three, and four; PHQ-9 antepartum group: two, four, and five; PHQ-9 postpartum group: one, five, and six.

### Exploratory factor analysis (EFA)

For both the EPDS and PHQ-9, we ran EFAs comparing the two models suggested by parallel analyses (i.e., two-factor and three-factor models) using the entire sample, the antepartum sample, and the postpartum sample (see Table 2).

**Table 2:** Item-level exploratory factor analyses of the Edinburgh Postnatal Depression Scale (EPDS) and of the Patient Health Questionnaire-9 (PHQ-9)

	Items	Two-factor models						Three-factor models								
		Entire sample		Antepartum sample		Postpartum sample		Entire sample			Antepartum sample			Postpartum sample		
		F1	F2	F1	F2	F1	F2	F1	F2	F3	F1	F2	F3	F1	F2	F3
EPDS	1. Laugh	**.83**	.00	**.82**	−.04	**.78**	.04	.55	.04	**.58**	.01	**.88**	.03			
	2. Enjoyment	**.80**	−.05	**.78**	−.10	**.73**	.03	.29	−.03	**.89**	.09	**.74**	−.03			
	3. Self-blame	.32	**.41**	.31	**.39**	−.06	**.78**	.31	**.35**	.12	.20	.15	**.38**			
	4. Anxious	−.04	**.81**	−.94	**.83**	.18	**.55**	−.05	**.79**	.02	−.02	−.07	**.83**			
	5. Scared	.04	**.76**	.33	**.74**	.20	**.55**	.10	**.75**	−.01	.02	.03	**.74**			
	6. Hard to cope	.32	**.36**	.30	**.37**	.03	**.60**	.14	**.26**	.12	.10	.21	**.39**			
	7. Hard to sleep	**.65**	.14	**.62**	.13	**.63**	.17	**.73**	.10	.24	**.53**	.19	.06			
	8. Sad	**.83**	.08	**.78**	.09	**.67**	.27	**.81**	.02	.36	**.57**	.31	0.04			
	9. Crying	**.72**	.15	**.70**	.15	**.67**	.21	**.80**	.09	.26	**.90**	.02	−.02			
	10. Self-harm	**.50**	.19	**.40**	.26	**.45**	−.06	**.52**	.14	.18	**.57**	−.05	.16			
PHQ-9	1. Anhedonia	**.44**	.42	**.47**	.42	.62	.36	**.43**	.42	.05	**.45**	.42	.04	**.46**	.17	.27
	2. Depressed mood	**.68**	.10	**.63**	.13	.91	.17	**.76**	.15	−.10	**.67**	.17	−.06	**.92**	−.11	.11
	3. Sleeping difficulties	−.18	**.76**	−.22	**.83**	.26	.18	−.16	**.74**	.20	−.22	**.82**	.01	.00	.01	**.84**
	4. Fatigue.	.16	**.57**	.14	**.59**	.40	.37	.17	**.59**	−.01	.17	**.62**	−.05	.21	.24	**.34**
	5. Appetite changes	.30	**.42**	.32	**.38**	.47	.48	.19	**.35**	.23	.23	**.33**	.18	.29	**.37**	.17
	6. Feeling of worthlessness	**.89**	−.68	**.81**	−.02	.74	.49	**.85**	−.06	.74	**.80**	−.01	.01	**.75**	.28	−.09
	7. Concentrations difficulties	**.52**	.21	**.46**	.27	.31	.70	.28	.08	**.46**	.29	.16	**.35**	.07	**.77**	−.03
	8. Psychomotor agitation	**.46**	.26	**.42**	.24	.23	.87	.00	.03	**.86**	−.01	−.02	**.96**	−.01	**.84**	.11
	9. Suicide ideation	**1.00**	−.06	**1.09**	−.20	.70	.52	**.88**	−.07	.16	**1.04**	−.19	.06	**.58**	.35	.06

Bold fonts indicate items’ scale assignments

The antepartum sample consists of 1166 pregnant people, while the postpartum sample consists of 311 people who gave birth to one or more children in the 6 months prior to the time of data collection. The entire sample includes both antepartum and postpartum samples

Those reported in the table are the average loadings across the various factor extraction and rotation methods performed by the R package EFAtools (Steiner & Grieder 2020)

Regarding EPDS, eigenvalues and percentage cumulative variance were as follows: 3.74 (37.4%) and 1.94 (56.8.0%) for the entire sample’s two-factor solution; 3.36 (33.5%) and 1.87 (52.2%) for the antepartum group’s two-factor solution; 3.01 (30.9%) and 2.10 (51.9%) for the postpartum group’s two-factor solution; 3.17 (31.7%), 1.73 (49.0%), and 2.16 (60.6%) for the entire sample’s three-factor solution; 2.17 (21.7%), 1.81 (38.8%), and 1.73 (56.1%) for the antepartum group’s three-factor solution; lastly, EFA could not be estimated for the postpartum group’s three-factor model. Item 6 does not load on any of the extracted factors within the antepartum group.

Regarding the PHQ-9, eigenvalues and percentage cumulative variance were as follows: 3.29 (36.5%) and 1.62 (56.4%) for the entire sample’s two-factor solution; 3.10 (34.4%) and 1.61 (52.3%) for the antepartum group’s two-factor solution; 3.60 (40.0%) and 3.15 (75.0%) for the postpartum group’s two-factor solution; 2.71 (30.1%), 1.41 (45.7%), and 1.29 (60.0%) for the entire sample’s three-factor solution; 2.72 (30.2%), 1.48 (46.6%), and 1.20 (59.9%) for the antepartum group’s three-factor solution; 2.63 (29.2%), 2.24 (54.1%), and 1.28 (68.2%) for the postpartum group’s three-factor solution.

Table 3 presents CFA fit indices for the two- and three-factor models of the EPDS and PHQ-9 in the entire sample as well as the pre- and postpartum groups reported in Table 3.

**Table 3:** Confirmatory factor analysis indices of the two-factor and three-factor models of the Edinburgh Postnatal Depression Scale (EPDS) and Patient Health Questionnaire-9 (PHQ-9)

		*X*^2^ value	*df*	RMSEA	CFI	SRMR
EPDS	Entire sample					
	Two-dimensional model	161.754	34	.051	.992	.047
	Three-dimensional model	104.750	32	.039	.996	.039
	Antepartum sample					
	Two-dimensional model	142.547	34	.053	.987	.058
	Three-dimensional model	94.867	32	.041	.993	.048
	Postpartum sample					
	Two-dimensional model	53.758	34	.043	.998	.050
PHQ-9	Entire sample					
	Two-dimensional model	153.661	26	.058	.982	.063
	Three-dimensional model	100.885	24	.047	.989	.052
	Antepartum sample					
	Two-dimensional model	139.345	26	.064	.969	.084
	Three-dimensional model	97.436	24	.062	.982	.072
	Postpartum sample					
	Two-dimensional model	31.678	26	.027	.998	.048
	Three-dimensional model	21.102	24	.000	1.00	.041

The antepartum sample consists of 1166 pregnant people, while the postpartum sample consists of 311 people who gave birth to one or more children in the 6 months prior to the time of data collection. The entire sample includes both antepartum and postpartum samples

The items’ scale assignments are those indicated in Table 2 through the use of bold fonts

### Multigroup confirmatory factor analysis (CFA)

Table 4 shows fit statistics for multigroup confirmatory models with increasingly stringent equality constraints. Chi-squared difference tests are shown comparing each model to the one in the row above. For identification, the first item of each factor was set to 1 in each group. For the EPDS, baseline model parameters freely estimated for each group demonstrated acceptable fit [*X*^2^(68) = 196.306, CFI = .993, RMSEA = .051, SRMR = .056]. Constraining the free loadings to equality across groups did not significantly harm model fit (*p* = .58). Imposing further constraints on the estimated item thresholds did yield a significantly worse fitting model based on the chi-squared test (*p* < .001); however, overall fit based on other indices was still in the acceptable range (CFI = .989, RMSEA = .051, SRMR = .057). Similarly, for the PHQ-9, a baseline model showed acceptable overall fit [*X*^2^(52) = 181.023, CFI = .983, RMSEA = .058, SRMR = .076], and constraining loadings to equality across perinatal groups did not significantly worsen the fit (*p* = .12). Further constraining item thresholds to equality did significantly harm model fit compared to baseline (*p* < .001); however, other fit indices remained within acceptable limits. Thus, both the EPDS and PHQ-9 demonstrated evidence of at least weak measurement invariance across perinatal groups using the two-factor models.

**Table 4:** Fit statistics and likelihood ratio tests of equality constraints across perinatal groups for two-factor models

	Equality constraints	CFI	RMSEA	SRMR	*X*^2^ value	*df*	*X*^2^ diff*.*	*df* diff*.*	*p*
EPDS	None	.993	.051	.056	196	68	-	-	-
	Loadings	.992	.048	.058	207	76	6.62	8	.58
	Loadings & thresholds	.989	.051	.057	276	94	163	18	< .001
PHQ-9	None	.983	.058	.076	181	52	-	-	-
	Loadings	.981	.058	.078	205	59	11.3	7	.12
	Loadings & thresholds	.968	.067	.076	324	75	260	16	< .001

### Reliability

Both scales performed similarly across measures of reliability and internal consistency, though the EPDS showed slightly higher ratings across all metrics. Scores on both scales had adequate alphas (.80 and .84 for PHQ-9 and EPDS, respectively) and similarly high overall reliable variance (omega total) based on a two-factor hierarchical model (Revelle & Condon 2019). Compared to the PHQ-9, the EPDS showed higher omega hierarchical (.67 versus .57). Finally, the average inter-item correlation was higher for the EPDS Table 5.

**Table 5:** Reliability statistics for the EPDS and PHQ-9 scores

Scale	Alpha	Omega total	Omega hierarchical	Average item correlation
EPDS	.84	.86	.65	.35
PHQ-9	.80	.82	.57	.30

## Discussion

### Comparison with previous studies

The results presented in this study supported a two-factor solution for both scales across perinatal samples. However, while the EPDS performs well in both the antepartum and postpartum groups in terms of factor model fit and reliability (alpha, omega, and average item correlation), the PHQ-9 shows adequate performance only in the antenatal group and has inconsistent factor loadings and poor model fit in the postpartum group. Therefore, our findings indicate that the PHQ-9 may not be well-adapted for measuring depressive symptoms in the postpartum Italian-speaking population and that the EPDS should be preferred. For both scales, however, caution is required when comparing antepartum to postpartum scores, as discussed below. Lastly, given that the general factor heavily saturates the individual factors in both scales, the EPDS and PHQ-9 should probably be used as single-factor scales.

The two-factor structure model of the EPDS was consistently observed in the whole sample (without using residual covariances) as well as separately in the antepartum and postpartum samples. The two factors detected were related to depression and anxiety symptoms, respectively. Invariance testing revealed that loadings can be equated across antepartum and postpartum but not the thresholds. This suggests that although the EPDS items are related to the construct of depressive symptomatology in a similar way, one should take caution in interpreting mean differences across antepartum and postpartum groups. On a practical level, this means that a score of *X* at prepartum does not necessarily indicate the same level of depressive symptoms as a score of *X* at postpartum, but a change of ±*Y* points likely indicates the same change in both groups.

Our results concerning the factor structure of the EPDS are in line with the only previous Italian study on the topic (Della Vedova et al. 2022). However, they are inconsistent with most of the international literature which has found a three-factor solution (e.g., Coates et al. 2017; Kubota et al. 2018; Long et al. 2020). Differences in factor number and composition may plausibly depend on differences in cultural and/or language features. In fact, culturally sensitive cut-off values for the EPDS have been recommended, and they vary considerably, ranging from nine to fourteen for different populations (Halbreich & Karkun 2006; Smith-Nielsen et al. 2018). Such differences are likely owing to cultural variations in the attributions and expressions of depressive symptoms and the language used to describe them (Haroz et al. 2017; Lara-Cinisomo et al. 2020).

Regarding PHQ-9, our findings suggest a two-factor structure model in the antenatal group. Unlike the EPDS, only very few studies have thus far investigated the factor structure of the PHQ-9 in perinatal samples. Different factor structures were found during the antepartum period, and it seems plausible that these differences stem from cultural differences. Two studies involving Peruvian pregnant women agreed on indicating the same two-factor solution with the same items assigned to each scale (Smith et al. 2022a,b; Zhong et al. 2014). Similarly, a Japanese study found a two-factor model but with different assignments of items to scales (Wakamatsu et al. 2021). Further two studies involving Ethiopian versus Ivorian and Ghanaian pregnant women suggested a one-factor structure (Barthel et al. 2015; Woldetensay et al. 2018). Finally, a three-factor model (cognitive-affective, somatic, and pregnancy-related) was considered adequate to screen depression in Spanish pregnant women (Marcos-Nájera et al. 2018). To our knowledge, no studies except ours have examined the factor structure of the PHQ-9 in postpartum samples.

A recent systematic review and meta-analysis on screening for perinatal depression identified 15 studies providing psychometric comparisons between the EPDS and PHQ-9 and found that their operating characteristics of sensitivity, specificity, and area under the curve were remarkably similar (Wang et al. 2021). However, this study focused on the diagnostic accuracy of these scales rather than their psychometric properties. The present study offers important new evidence about the measurement invariance of these scales across the perinatal period which can inform the choice of which scale to use in clinical practice and research.

The different performances observed between the PHQ-9 and EPDS, especially in the postpartum group, support a possible partial explanation that they capture partially distinct features of depressive symptomatology. In fact, growing evidence indicates that genetic etiologies for perinatal depression overlap only partially with those for non-perinatal depression (Viktorin et al. 2016) and that there exist different types and severities of perinatal depression (Putnam et al. 2017). Only depression occurring in the later postpartum period (i.e., after the 8th week postpartum) seems to be more similar to a major depressive disorder occurring outside of the perinatal period (Batt et al. 2020). It is therefore possible that the main differences are likely related to the specific development of the two scales. The EPDS was specifically devised for postpartum depression using items drawn from three scales for anxiety and depression [i.e., the Irritability, Depression, and Anxiety Scale (Snaith et al. 1978), the Hospital Anxiety and Depression Scale (Zigmond & Snaith 1983), and the Anxiety and Depression Scale (Bedford et al. 1976)], and deemphasizing the somatic symptoms that might overlap with depressive symptoms even when they should be considered normative during postpartum. The PHQ-9 was instead developed specifically to identify depressive disorders based on DSM-IV criteria and was derived from the Primary Care Evaluation of Mental Disorders (PRIME-MD; Spitzer et al. 1994), which was originally devised to identify mood, anxiety, somatoform, alcohol, and eating disorders in the general population. As a result, in both scales, some items are not entirely consistent with the depressive dimension; the PHQ-9 includes items addressing somatic symptoms, whereas the EPDS includes items addressing anxiety. This is a key difference because, on the one hand, somatic symptoms are strongly experienced by perinatal women, even if they are not clinically depressed (Pereira et al. 2014), and the presence of somatic symptoms during antenatal depression predicts postpartum depressive symptoms even if these symptoms have subsided (Roomruangwong et al. 2017). On the other hand, besides depressive symptoms, anxiety is the most common psychological symptom observed in both pregnant people and new mothers (Cena et al. 2020; Cena et al. 2021a, 2021b; Nakić Radoš et al. 2018).

### Strengths and limitations

The strengths of the present study include the use of a large perinatal sample and several clinical centers located throughout Italy. Furthermore, this study used multigroup confirmatory factor analysis to assess measurement invariance across the perinatal period—the first paper that we know of to apply this modern psychometric approach to compare the EPDS and PHQ-9. Finally, this is the first study to examine the factor structure of the Italian version of the EPDS in an antepartum sample, as well as the first to examine the factor structure of the Italian version of the PHQ-9 in a perinatal sample. However, there are also some noteworthy limitations. Firstly, the cross-sectional design precludes the evaluation of the test-retest reliability of the scales. Another limitation regards the fact that the factor structure of both the EPDS and the PHQ-9 across trimesters was not examined. Lastly, because our sample population was entirely composed of people living in Italy, it may not be representative of other country populations.

## Conclusion

In conclusion, in the present study, the Italian version of the EPDS demonstrated reliability but weak (i.e., factor loadings equated) measurement invariance across antepartum and postpartum groups. In contrast, the Italian version of the PHQ-9 showed adequate performance with pregnant people but had inconsistent factor loadings and poor model fit with postpartum people. Therefore, we conclude that the EPDS should be preferred to the PHQ-9 for measuring depressive symptoms in the perinatal population but should be used with caution when comparing antepartum to postpartum scores. Lastly, we recommend that both the EPDS and PHQ-9 can be used as a single-factor scale.

## Data Availability

The data that support the findings of this study are available from the corresponding author upon reasonable request.
